# Editorial: Food/Diet Supplements From Natural Sources: Current Status and Future Challenges From a Pharmacological Perspective

**DOI:** 10.3389/fphar.2021.817606

**Published:** 2021-12-16

**Authors:** Michał Tomczyk, Marcello Locatelli, Sebastian Granica

**Affiliations:** ^1^ Department of Pharmacognosy, Medical University of Bialystok, Bialystok, Poland; ^2^ Department of Pharmacy, University of Studies G. d’Annunzio Chieti and Pescara, Chieti, Italy; ^3^ Department of Pharmacognosy and Molecular Basis of Phytotherapy, Warsaw Medical University, Warsaw, Poland

**Keywords:** extraction techniques, chemometric approaches, bioactives, food supplements, diet supplements, herbal medicine, hyphenated instrument configurations, ethnopharmacology

Nowadays, several natural products are used as food additives even if knowledge on their properties is not complete. Often, several techniques can be employed to improve extractions, workup, and isolation/purification of bioactive materials from natural sources. These findings are reported well and exposed in Dall’Acqua et al., Ahmad et al., and Li et al. These authors report new extraction procedures and chemical profiles able to justify specific biological activities with the phytocomplex. From an industrial point of view, the availability of procedures and knowledge (firstly developed in laboratory scale) is essential to obtain the scale-up and adequate quality control, particularly to the innovative instrument configurations able to improve not only the analytical performances (especially sensitivity and selectivity) but also to reduce solvents consumption, time-per-analysis, and ruggedness, following the GAC (Green Analytical Chemistry) guidelines. Specifically, this research topic focuses on the biological activities of a specific plant-derived material and the discovery of innovative activities and new biological targets. In this field, more interesting are the papers from Zhang et al., Jin et al., Blažević et al., Chang et al., Schreck and Melzig. Furthermore, in Pharmacology and Ethnopharmacology, this collection focuses on specific effects ideally on identifiable targets. Specifically, some papers report interesting approaches/applications of natural products on health protection, such as Wang et al., Ye et al., Jiang et al., and Yong et al. This research topic also includes works on food supplements (Chen and Tsim) and an interesting paper related to a very recent problem related to the naturally derived products that are not fully identified and regulated by current legislation, especially concerning heavy metals (Puścion-Jakubik et al.). Other essential elements in this topic collection are two review papers, the first on the trends of adulterated and illegal food supplements in the EU based on the warnings of the Rapid Alert System for Food and Feed (Koncz et al.) and the second on the potential of edible and herbal plants for the prevention and management of COVID-19 (Li et al.).

